# Pseudoaneurysm of the Popliteal Artery After (Revision) Knee Arthroplasty

**DOI:** 10.1016/j.artd.2021.11.002

**Published:** 2021-12-02

**Authors:** Biko A. Schermer, Arne C. Berger, Wouter Stomp, Joris C.T. van der Lugt

**Affiliations:** a Department of Orthopaedics, Reinier Haga Orthopedic Center, Zoetermeer, the Netherlands; b Department of Surgery, Amphia Hospital, Breda, the Netherlands; c Department of Radiology, LangeLand Hospital, Zoetermeer, the Netherlands; d Department of Radiology, Haga Hospital, The Hague, the Netherlands; e Department of Orthopaedics, Haga Hospital, The Hague, the Netherlands

**Keywords:** Pseudoaneurysm, Popliteal artery, Total knee arthroplasty, Revision knee arthroplasty

## Abstract

Pseudoaneurysm of the popliteal artery is a rare complication of total knee arthroplasty (TKA), with a reported incidence of 0.0095% to 0.088%. We describe the case of a 66-year-old female who underwent conversion of unicompartmental knee arthroplasty (2014) to a TKA because of instability symptoms. A pseudoaneurysm of the popliteal artery was found postoperatively on ultrasound performed because of persistent symptoms of pain and tightness of her calf and hypesthesia of digits 3 to 5. She was treated endovascularly with placement of a covered stent. At the most recent follow-up (8 months after surgery), the complaints of hypesthesia persist. A pseudoaneurysm of the popliteal artery is a rare, yet well-described, complication of TKA often found coincidentally on Duplex ultrasound usually performed to rule out a deep venous thrombosis. Prompt diagnosis is of great importance given the potential to developing compartment syndrome or irreversible neurological deficits.

## Introduction

Pseudoaneurysms of the popliteal artery are a rare complication after total knee arthroplasty (TKA), with reported incidence varying between 0.0095% and 0.088% [[1], [2], [3], [4], [5], [6], [7]]. In a recent, large systematic review, pseudoaneurysm of the popliteal artery was found to be the most common type of vascular injury after TKA (45%), followed by occlusion (33%) and transection (14%) [8]. A pseudoaneurysm, or false aneurysm, is a contained local hematoma in direct connection with an artery due to a defect in the layers of the arterial vessel wall. This is in contrast to a true aneurysm where all layers of the vessel wall are intact. Often, a pseudoaneurysm of the popliteal artery presents similar to a deep venous thrombosis (DVT): progressive swelling, pain, and tightness of the calf. If not recognized early, it has the potential to developing compartment syndrome or irreversible neurological deficits.

In this case report, we describe the case of a 66-year-old female who underwent revision knee arthroplasty due to instability symptoms of her unicompartmental knee arthroplasty. On postoperative day 4, Duplex ultrasound—made because of hindering symptoms and to exclude a DVT—showed a pseudoaneurysm of the popliteal artery, which was treated endovascularly with placement of a covered stent. The patient provided informed consent for publication of this case report.

## Case history

**Figure 1:**
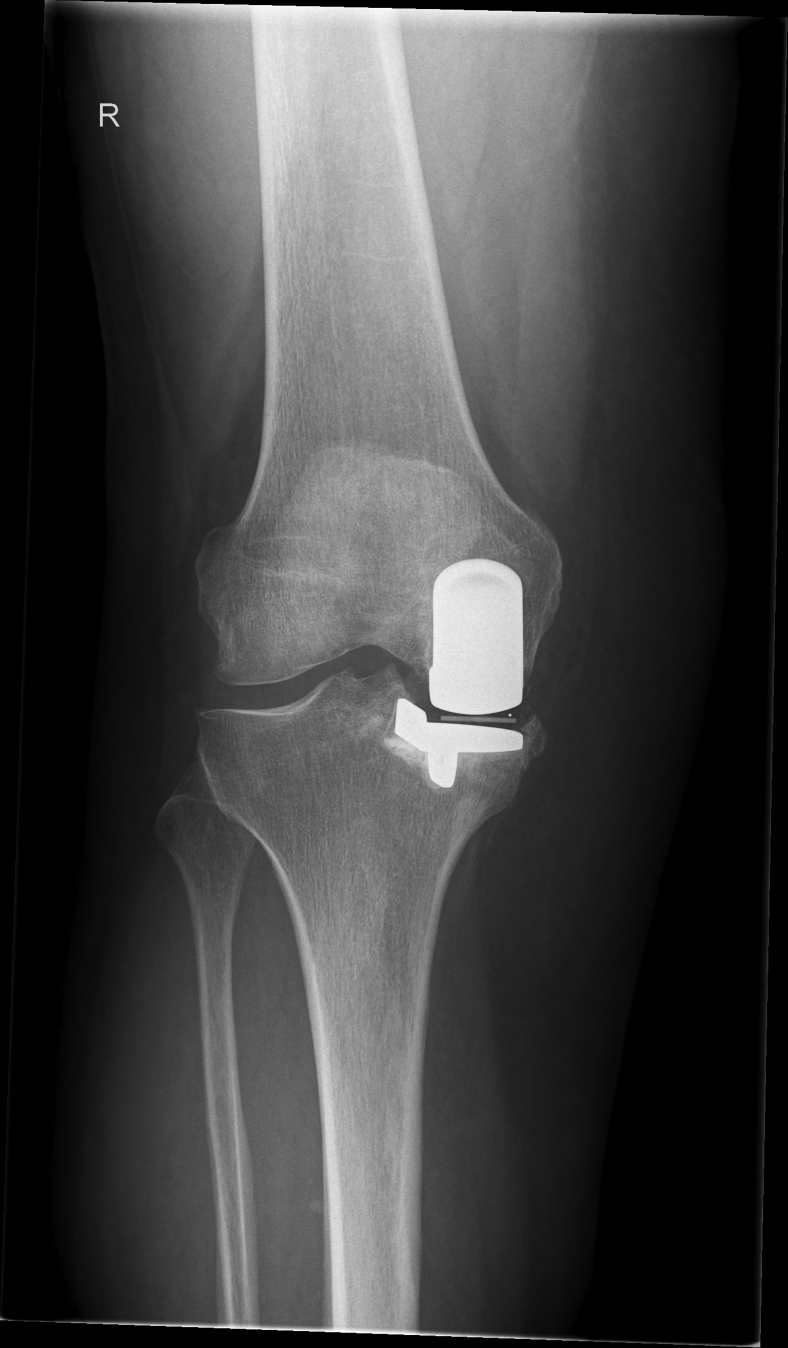
Preoperative anteroposterior radiograph showing the unicompartmental knee arthroplasty.

**Figure 2:**
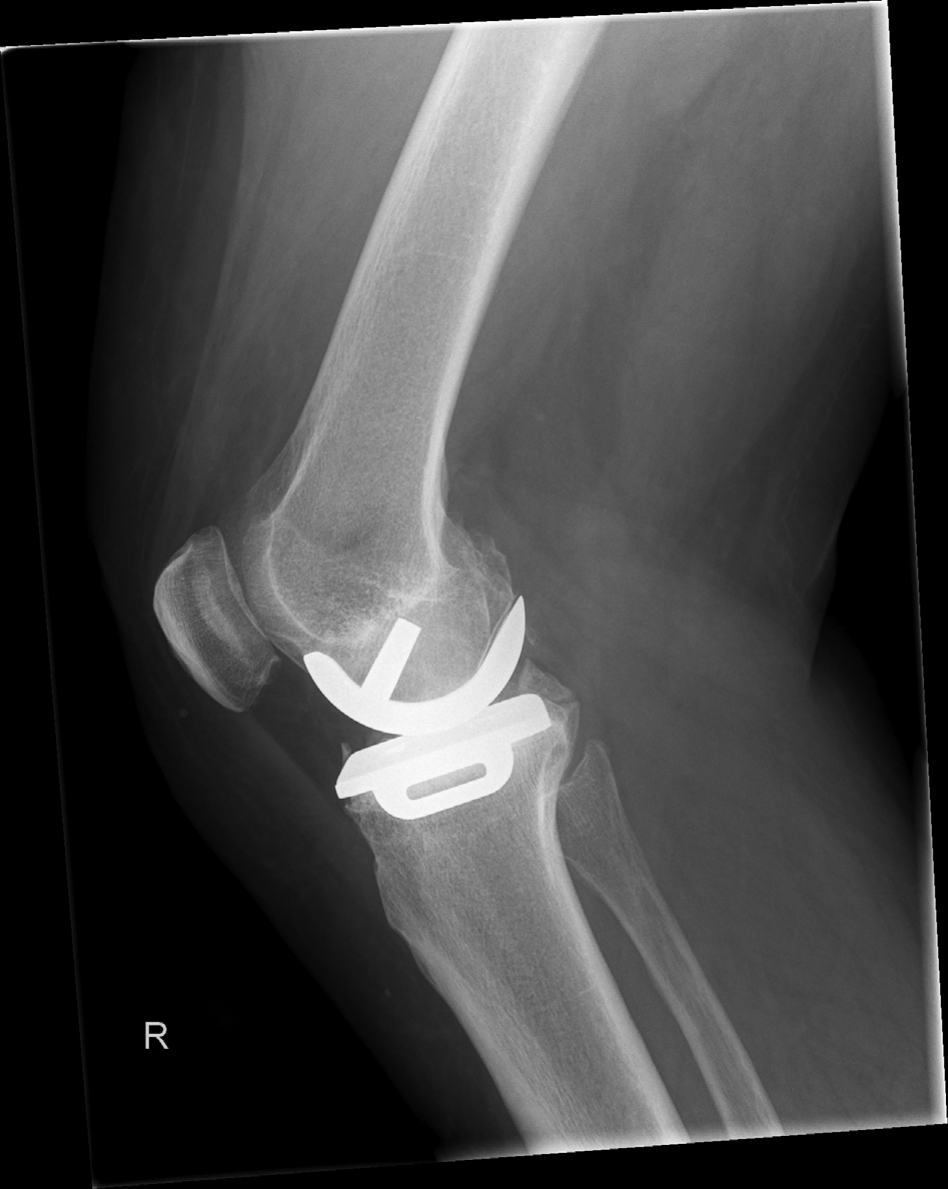
Preoperative lateral radiograph showing the unicompartmental knee arthroplasty.

A 66-year-old female patient with a unicompartmental knee arthroplasty (2014) (Figure 1, Figure 2) underwent conversion to a TKA because of anteroposterior instability symptoms, which she developed after a fall from the stairs 1.5 years before the revision. The patient’s medical history includes asthma, hypertension, type 2 diabetes mellitus, and obesity (body mass index of 36 kg/m^2^). Preoperative physical examination showed a grade 3 positive Lachman test, suggesting an anterior cruciate ligament rupture and a positive posterior sag sign indicative of concomitant posterior cruciate ligament damage. Varus and valgus stress tests showed no signs of instability; neurological examination was without abnormalities.

**Figure 3:**
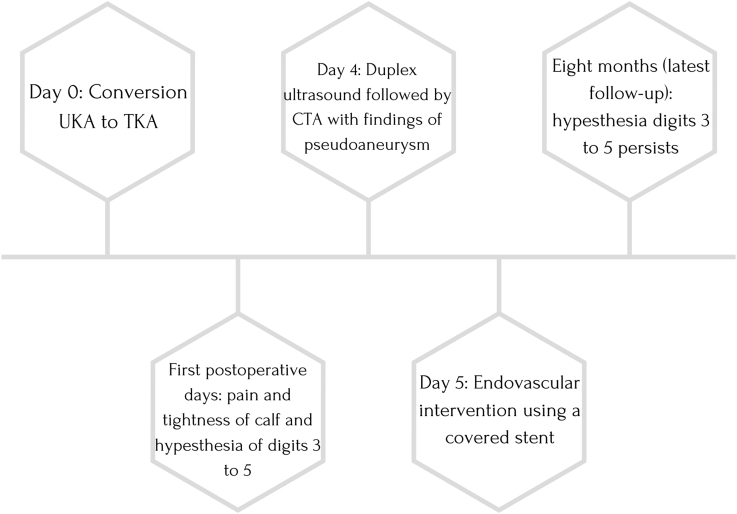
Timeline. CTA, computed tomography angiography; UKA, unicompartmental knee arthroplasty.

**Figure 4:**
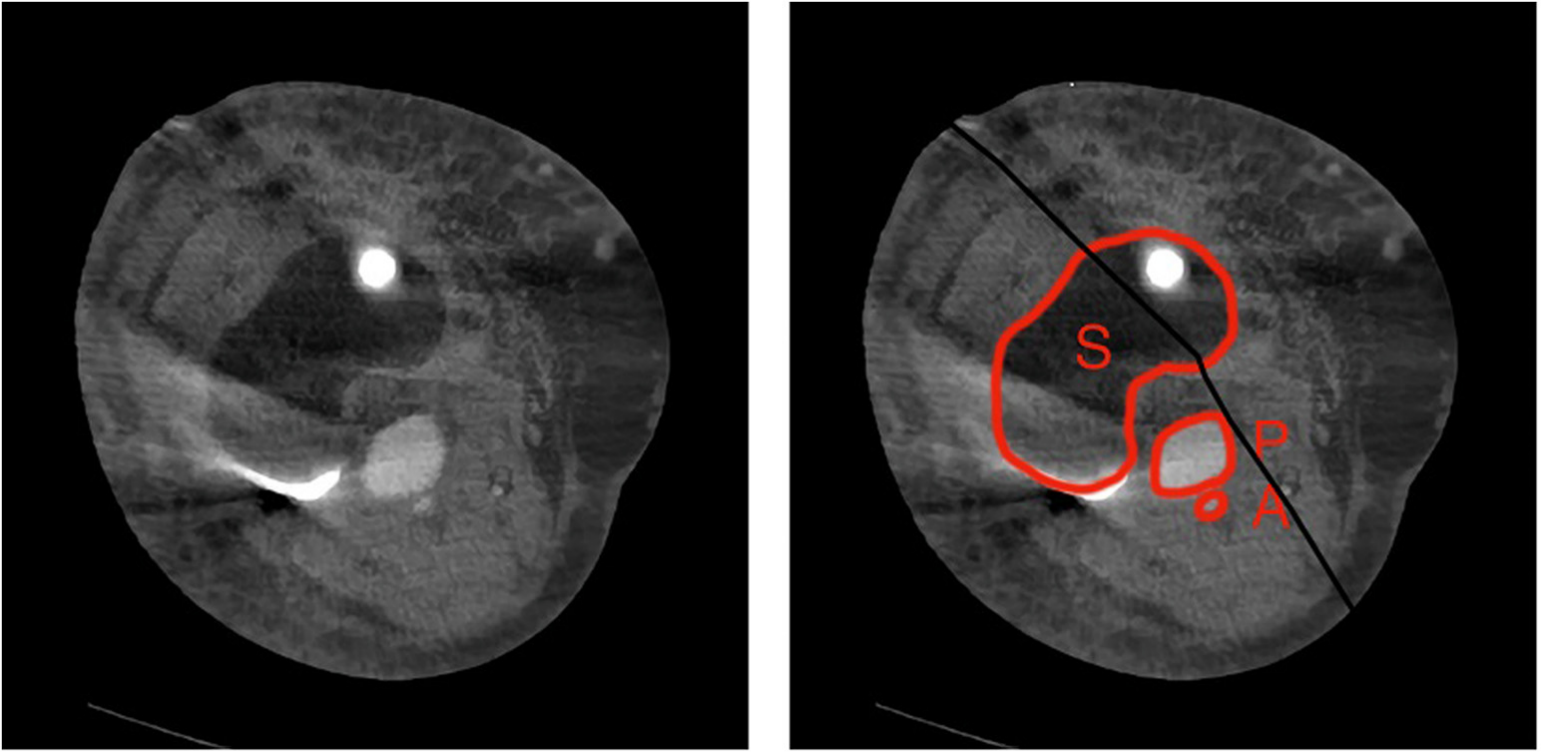
Preoperative axial CT angiography image at the level of the joint space showing the pseudoaneurysm directly ventral of the popliteal artery. A, popliteal artery; P, pseudoaneurysm; S, spacer of the knee prosthesis.

**Figure 5:**
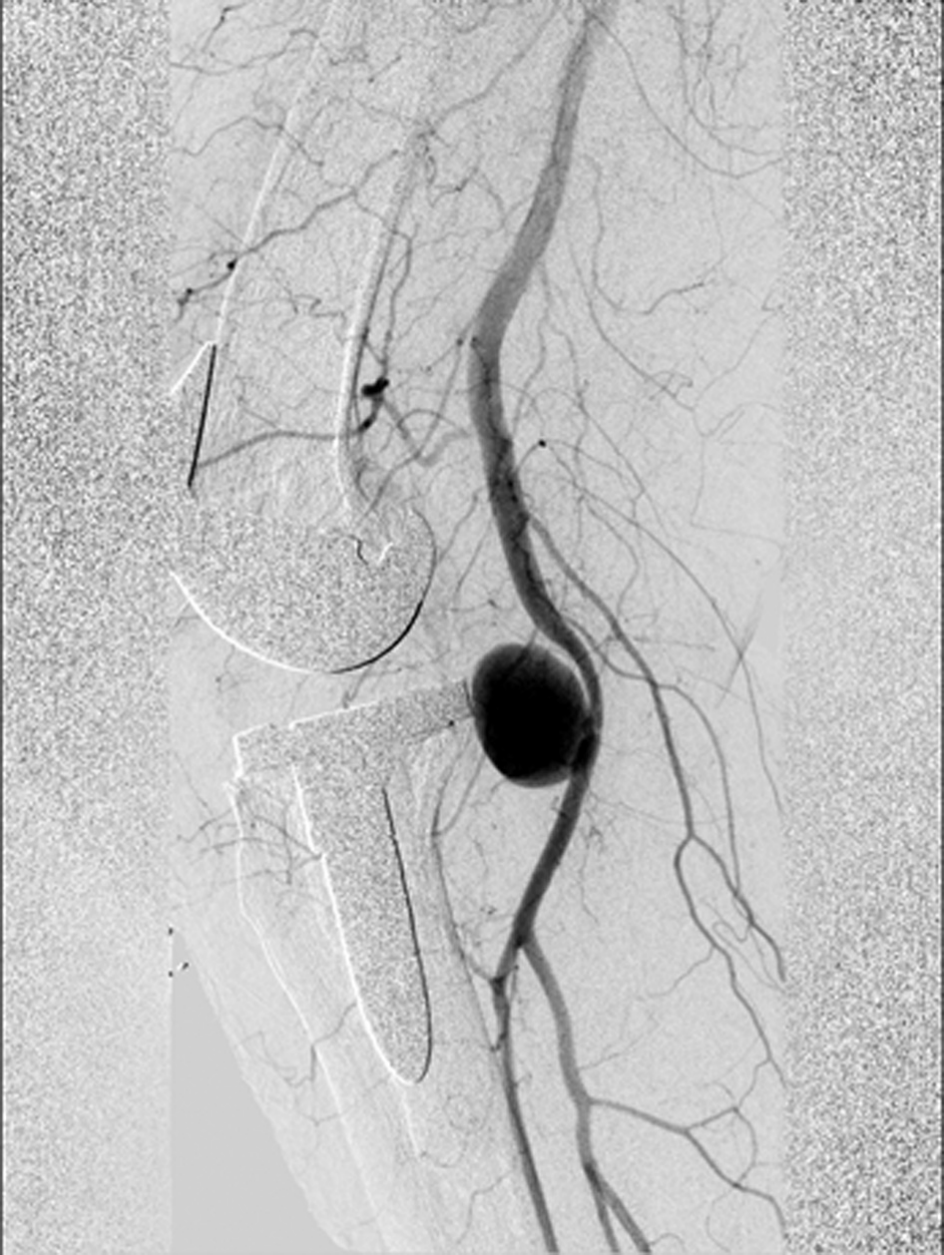
Sagittal angiography image showing the pseudoaneurysm originating from the popliteal artery, directly dorsal of the tibial part of the total knee arthroplasty.

**Figure 6:**
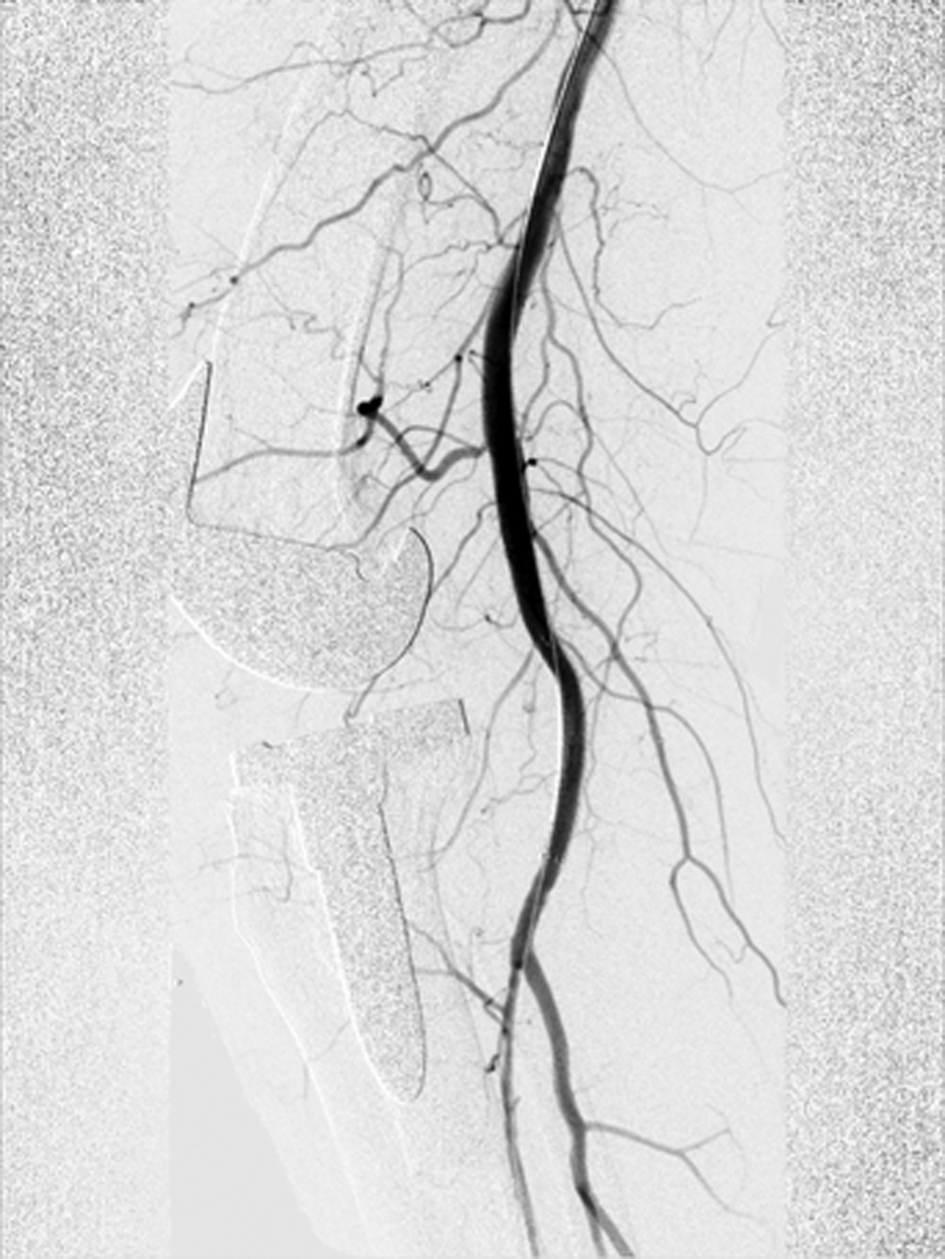
Sagittal angiography image acquired directly after placement of the stent graft showing complete exclusion of the pseudoaneurysm from the arterial circulation.

**Figure 7:**
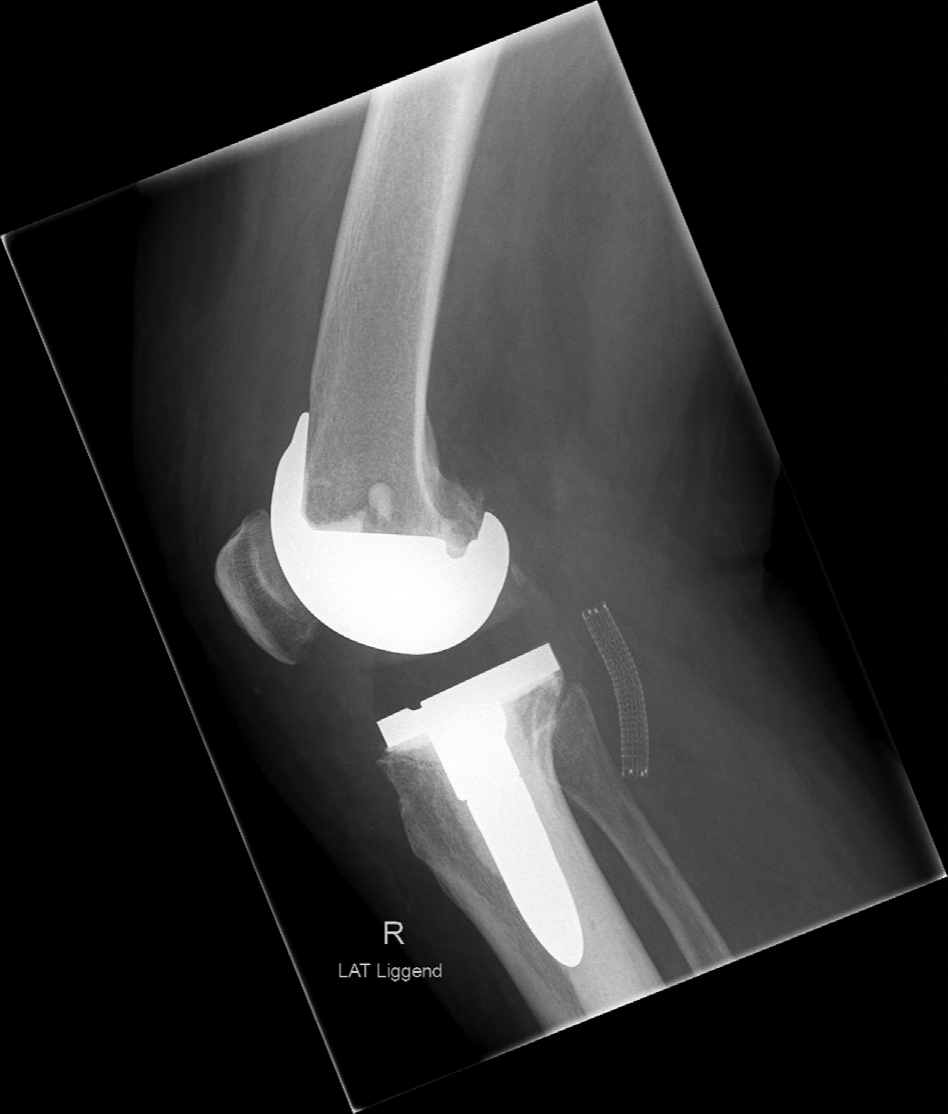
Postoperative sagittal radiograph after treatment showing the position of the stent graft.

During surgery, a cemented revision TKA with a short-stemmed tibial component was placed (type: Triathlon PS Cemented; Stryker, Amsterdam, the Netherlands). The surgery was performed without intraoperative complications, and no tourniquet was used. Furthermore, local infiltration anesthesia was applied in the posterior and anterior capsules and subcutaneously. On postoperative day one, the radiographs (anteroposterior and lateral) showed proper position of the prosthesis with no signs of complications; the hemoglobin level was 6.9 mmol/L (ref 7.2-9.5), down from a preoperative level of 8.9 mmol/L. On postoperative day 2, the patient experienced pain in the calf and the knee and decreased sensibility of the lateral edge of the foot. Physical examination showed a tense calf and foot (not red, not shining). Palpation of the whole leg was painful, including the calf. Moreover, there was decreased sensibility of the dorsal side of toes 3-5 and over the lateral edge of the foot, which the patient had noticed after spinal anesthesia had worn off. All motor functions were intact (dorsiflexion, plantar flexion, eversion and inversion of the ankle joint, flexion and extension of the toes). On postoperative day 4, a hematoma of the operative leg was noted. The clinical presentation seemed to best fit an expanding hematoma. Because of hindering symptoms and to exclude a DVT, a venous Duplex ultrasound of the lower leg was performed on the fourth postoperative day (Fig. 3). On the ultrasound, no DVT was identified. However, a globular abnormality in direct relation to the popliteal artery with arterial pulsations was found. Computed tomography angiography (CTA) confirmed the presence of a pseudoaneurysm at the level of the joint space of the knee prosthesis originating from the popliteal artery with a diameter of approximately 22 mm (Fig. 4). The popliteal artery itself was patent. Owing to a wide neck of the pseudoaneurysm (1-2 cm), neither thrombin injection nor coiling was possible given the risk of distal embolization. Also, there is a chance that bleeding might persist after coiling of the pseudoaneurysm. Thereupon, after consultation with the interventional radiologist and the vascular surgeon, it was decided to treat the pseudoaneurysm endovascularly with a covered stent (Fig. 5). Through an antegrade sheath in the common femoral artery, a 7 × 50-mm covered stent (type: Viabahn Endoprosthesis; W.L. Gore & Associates, Flagstaff, AZ) was placed; subsequent angiography showed complete elimination of the pseudoaneurysm (Fig. 6). The procedure was uncomplicated, and postprocedural dual antiplatelet therapy was started for 6 months (Fig. 7). On day 7, the patient was discharged in good condition, with residual symptoms of pain, swelling, and hypesthesia of the dorsal side of digits 3 to 5. The stent was patent at control Duplex ultrasound after 2 weeks.

In outpatient follow-up, an ultrasound of the lower leg and magnetic resonance imaging (MRI) of the lumbar spine were performed because of persistent paresthesia (neuropathic pain in the lower leg and foot and tingling of digits 3 to 5) and hypesthesia of the same toes, lateral edge of the foot, and the heel. However, no abnormalities were found. Also, a neurologist was consulted who attributed the patient’s symptoms to tibial nerve neuropathy—due to compression of the pseudoaneurysm—which was confirmed on electromyography. After contacting the clinical neurophysiologist, it appeared to involve a mild compression neuropathy, which would likely recover without permanent symptoms within 1 year. At the most recent follow-up (8 months after surgery), the patient showed good, yet slower than normal, functional recovery; the complaints of hypesthesia of digits 3 to 5 of the right foot and heel persisted.

## Discussion

In current literature, an incidence of 0.0095% to 0.088% has been reported for pseudoaneurysms of the popliteal artery after TKA [[1], [2], [3], [4], [5], [6], [7]]. Nearly all reports describe progressive swelling, posterior knee pain, and tightness of the calf as the initial clinical symptoms. In addition, formation of posterior hematoma, a pulsatile mass, and an audible souffle have been described, which may contribute to the differentiation between a DVT and a pseudoaneurysm [3]. In the series by Ammori et al., 2 patients presented with a compartment syndrome, for which fasciotomy was performed, before the diagnosis of pseudoaneurysm was identified [1]. Bernhoff et al. reported the largest group in the literature to date, and in their series of eleven patients, one patient presented with a compartment syndrome and one patient with recurrent hemarthrosis [4]. Patients with recurrent hemarthrosis are also described in the series of Boutchichi et al. [2]. In addition, neurological symptoms including hypesthesia of the foot localized in the first web space or on the plantar side may be present [2,3].

The majority of postoperative pseudoaneurysms are diagnosed during ultrasound imaging performed because of clinical suspicion of a DVT [1,3,5]. In addition, CTA can be performed to confirm the diagnosis and for treatment planning. Alternatively, an MRI scan can be performed to confirm the diagnosis. However, both CTA and MRI often show artifact formation due to the prosthesis [2,9]. In literature, the interval between occurrence of the complication (intraoperatively) and diagnosis is remarkably long. Ammori et al. found a median interval of 15 days (range: 7-27) [1]. In the series by Bernhoff et al., a pseudoaneurysm was diagnosed in 11 patients with a median interval to diagnosis of 41 days (range: 2-90) [4]. Compared with the literature, the diagnosis in our case was found relatively early (4 days postoperatively) on ultrasound. This ultrasound was mainly performed because of hindering symptoms and to exclude a DVT; a bleed was not directly suspected. Because of the early presentation and persistent symptoms that hindered clinical recovery, the pseudoaneurysm was identified quickly. It involved an expeditious workup with ultrasound followed by CTA for confirmation of the diagnosis and for planning of treatment. Considering the current review of the literature, the additional diagnostic tools in outpatient follow-up could have been reduced as the symptoms can be explained by the pseudoaneurysm.

Small pseudoaneurysms with a narrow neck can be resolved by ultrasound-guided compression therapy or local thrombin injection [10,11]. In most cases, however, surgical repair or endovascular treatment by means of placement of a covered stent is necessary. Advantages of endovascular treatment include its less invasive nature and a potentially reduced risk of infection of the prosthesis. Coiling of the pseudoaneurysm has been described in some cases; however, this does not completely preclude further bleeding. Remarkably, in the largest series, most patients were treated by open surgical intervention: for the studies by Bernhoff et al., Ammori et al., and Calligaro et al., respectively, 8 out of 11, 4 out of 7, and 3 out of 5 patients [1,4,5]. A possible explanation is that these studies looked at periods from 1987 onwards, when endovascular interventions were less feasible. When considering open surgical vs endovascular intervention, more recent studies show that endovascular interventions predominate nowadays [3,6,8,11]. In general, intervention took place 1 or 2 days after the radiological diagnosis [1,3].

After identification of the pseudoaneurysm in our patient, the cause was discussed between the orthopedic surgeon and the interventional radiologist. A preexisting pseudoaneurysm due to instability symptoms was considered unlikely, leaving intraoperative traction on the popliteal neurovascular structures in combination with scar tissue (due to revision surgery) as the causative factor. Several theories are described regarding the mechanism of injury to the popliteal artery during TKA. In general, indirect trauma is considered to be the causing factor. Injury to the popliteal artery during the tibial oscillating saw cut, placement of the retractor behind the tibial plateau, opening of the posterior capsule, or sacrificing the posterior cruciate ligament are indicated as possible causes as well [1,3,12]. Shin et al. suggest that the knee should be flexed when performing these potentially damaging maneuvers to move the popliteal neurovascular structures posteriorly [12]. In addition, these structures should be protected by well-placed retractors [12]. In general, these mechanisms of injury can be explained by their posterior location, which may lead to local manipulation of the popliteal neurovascular structures and traction through anterior translation of the tibia. Ammori et al. found that in 5 of 7 cases with a pseudoaneurysm of the popliteal artery, the posterior cruciate ligament had been sacrificed. However, they could not confirm this relation because of lack of data on the total number of patients with cruciate-sacrificing TKAs [1]. Furthermore, hyperextension is considered to be a possible mechanism, especially if atherosclerosis is present [1,3]. Boutchichi et al. in their case report of 3 patients mentioned the risk of injury during infiltration of local anesthetics; however, they concluded that this has never been described in English literature [2]. In revision surgery, a 2.4- to 2.7-times increased risk of acute arterial vascular injury is found, explained by fibrous scar tissue causing adhesions with the blood vessels making indirect injury easier during local manipulation [5,7,13].

Considering clinical implications of the pseudoaneurysm, compartment syndrome is particularly endangering [1,4]. At 1-year follow-up, Bernhoff et al. found functional limitations in 9 of eleven patients, which involved hypesthesia, weakness, pain and/or swelling [4]. Also, all eleven patients recovered more slowly than normal. In the study by Ammori et al., neuropathic pain was still present in 3 of 7 patients after 4 months of follow-up [1]. Hypesthesia of the foot, both dorsal and plantar, was also a residual symptom in a case reported by Geertsema et al. [3]. There have even been cases described in which the presence of the pseudoaneurysm led to a permanent foot drop, caused by compression on the common peroneal nerve [12,14]. In our patient, hypesthesia of digits 3 to 5 of the right foot and heel persisted at the most recent follow-up (8 months). The quick discovery and treatment of the pseudoaneurysm may have prevented more serious implications.

## Summary

A pseudoaneurysm of the popliteal artery is a rare, yet well-described, complication of TKA. Often, the pseudoaneurysm is found coincidentally on Duplex ultrasound, which is usually performed to rule out a DVT. Prompt diagnosis is of great importance given the potential vascular threat to the lower leg when a compartment syndrome develops, but also given the risk of irreversible neurological deficits because of common peroneal nerve or tibial nerve compression. Endovascular interventions offer good opportunities to repair the pseudoaneurysm in a minimally invasive manner.

## Learning points

- Pseudoaneurysm of the popliteal artery is a rare complication of knee arthroplasty, with an incidence between 0.01% and 0.1%.
- Indirect trauma with local manipulation intraoperatively is considered as a trauma mechanism.
- The clinical presentation is similar to that of a DVT, a pulsatile mass may be distinctive.
- Often observed coincidentally on ultrasound, CTA may be performed additionally for confirmation of diagnosis and planning of treatment.
- Usually, there is a delay in diagnosis: median intervals of 2 to 6 weeks after surgery.
- In the past, most cases were repaired with open surgery. Nowadays, endovascular intervention is preferred.
- Residual symptoms are not uncommon: hypesthesia, (neuropathic) pain, and/or swelling.

## Conflicts of interest

The authors declare that they have no known competing financial interests or personal relationships that could have appeared to influence the work reported in this article.

## Informed patient consent

Complete written informed consent was obtained from the patient for the publication of this study and accompanying images.

## Appendix A Supplementary data

Conflict of Interest Statement for Schermer

Conflict of Interest Statement for van der Lugt

Conflict of Interest Statement for Berger

Conflict of Interest Statement for Stomp
