# Supplementary material for: Pseudoaneurysm of the Popliteal Artery After (Revision) Knee Arthroplasty
Source: Arthroplast Today. 2021 Dec 2;13:1–6. doi: 10.1016/j.artd.2021.11.002 (PMC8649579; doi:10.1016/j.artd.2021.11.002)
Supplement: Conflict of Interest Statement for Schermer [file mmc1.docx]

# CONFLICT OF INTEREST STATEMENT

***American Association of Hip and Knee Surgeons***

(Adopted from the American Academy of Orthopaedic Surgeons disclosure statement)

The following form **must be filled out completely and submitted by each author (example, 6 authors, 6 forms).**

**All items require a response. If there is no relevant disclosure for a given item, enter "*None*.”**

Manuscript Title: *Pseudoaneurysm of the popliteal artery after (revision) TKA: a case report*

1. Royalties from a company or supplier (The following conflicts were disclosed)

*None*.

2. Speakers bureau/paid presentations for a company or supplier (The following conflicts were disclosed)

*None*.

3A. Paid employee for a company or supplier (The following conflicts were disclosed)

*None*.

3B. Paid consultant for a company or supplier (The following conflicts were disclosed)

*None*.

3C. Unpaid consultants for a company or supplier (The following conflicts were disclosed)

*None*.

4. Stock or stock options in a company or supplier (The following conflicts were disclosed)

*None*.

5. Research support from a company or supplier as a Principal Investigator (The following conflicts were disclosed)

*None*.

6. Other financial or material support from a company or supplier (The following conflicts were disclosed)

*None*.

7. Royalties, financial or material support from publishers (The following conflicts were disclosed)

*None*.

8. Medical/Orthopaedic publications editorial/governing board (The following conflicts were disclosed)

*None*.

9. Board member/committee appointments for a society (The following conflicts were disclosed)

*None*.

**Each author must sign AND print or type his/her name, date and submit a separate form**


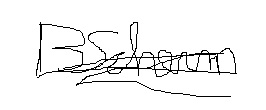
In addition, one BLINDED Conflict of Interest form (no author names used) should be submitted per manuscript with all author disclosures.

Biko Schermer 22-07-2021

Author Name (Print or Type) Author Signature Date
